# Sustained rheumatoid arthritis remission is uncommon in clinical practice

**DOI:** 10.1186/ar3785

**Published:** 2012-03-19

**Authors:** Femke HM Prince, Vivian P Bykerk, Nancy A Shadick, Bing Lu, Jing Cui, Michelle Frits, Christine K Iannaccone, Michael E Weinblatt, Daniel H Solomon

**Affiliations:** 1 Division of Rheumatology, Immunology and Allergy, Brigham and Women's Hospital, Harvard Medical School, Boston, MA, USA

## Abstract

**Introduction:**

Remission is an important goal of therapy in rheumatoid arthritis (RA), but data on duration of remission are lacking. Our objective was to describe the duration of remission in RA, assessed by different criteria.

**Methods:**

We evaluated patients from the Brigham and Women's Rheumatoid Arthritis Sequential Study (BRASS) not in remission at baseline with at least 2 years of follow-up. Remission was assessed according to the Disease Activity Score 28-C-reactive protein (DAS28-CRP4), Simplified Disease Activity Index (SDAI), and Clinical Disease Activity Index (CDAI) scores, and the recently proposed American College of Rheumatology (ACR)/European League against Rheumatism (EULAR) criteria for remission. Analyses were performed by using Kaplan-Meier survival curves.

**Results:**

We identified 871 subjects with ≥2 years of follow-up. Of these subjects, 394 were in remission at one or more time-points and not in remission at baseline, according to at least one of the following criteria: DAS28-CRP < 2.6 (*n *= 309), DAS28-CRP < 2.3 (*n *= 275), SDAI (*n *= 168), CDAI (*n *= 170), and 2010 ACR/EULAR (*n *= 158). The median age for the 394 subjects at entrance to BRASS was 56 years; median disease duration was 8 years; 81% were female patients; and 72% were seropositive. Survival analysis performed separately for each remission criterion demonstrated that < 50% of subjects remained in remission 1 year later. Median remission survival time was 1 year. Kaplan-Meier curves of the various remission criteria did not significantly differ (*P *= 0.29 according to the log-rank test).

**Conclusions:**

This study shows that in clinical practice, a minority of RA patients are in sustained remission.

## Introduction

Rheumatoid Arthritis (RA) is characterized by joint inflammation leading to joint destruction. This causes decreased functional capacity, work disability, and reduced quality of life [1]. Advances in the understanding of RA pathogenesis have led to the development of novel therapeutic targets and new treatment guidelines aiming for remission [2]. However, it is not generally accepted how best to define disease remission [3]. At least three remission definitions are in use: the Disease Activity Score (DAS)-28 < 2.6 and < 2.3 score, Simplified Disease Activity Index (SDAI) ≤3.3 score and Clinical Disease Activity Index (CDAI) ≤2.8 score [4-7]. Recently, the American College of Rheumatology (ACR), the European League against Rheumatism (EULAR), and the Outcome Measures in Rheumatology Initiative (OMERACT) developed new remission criteria [8,9], the ACR/EULAR provisional definition of remission. All of these remission definitions examine disease activity at a single point in time, making them less useful for long-term follow-up studies [10,11]. Studies evaluating tight control and treat-to-target strategies advocate that remission should be reached as soon as possible and should be maintained during the course of the disease [12,13]. The investigators suggest that remission must be sustained to halt joint damage [12,13]. Relatively little is known about the duration of remission in clinical practice. With remission being a stated goal of RA treatment, it would thus be important to compare remission criteria and to examine the duration of RA remission [2].

To understand remission duration in RA, we examined a large clinical cohort of patients followed up for multiple years. Our aims were (a) to describe the duration of remission, regardless of how these patients achieved remission; and (b) to compare remission duration according to different remission criteria.

## Materials and methods

### The Brigham Rheumatoid Arthritis Sequential Study (BRASS) cohort

BRASS is a prospective, observational, single-center cohort with RA patients diagnosed by board-certified rheumatologists at the Brigham and Women's Hospital Arthritis Center [14]. Patients are prospectively monitored, and their RA is managed according to the preference of the treating rheumatologist. Patients complete a series of questionnaires every 6 months, and their rheumatologists carry out an annual structured physical examination with history, laboratory tests to determine RA activity, functional status, and adverse events. The study was approved by the Institutional Review Board for Brigham and Women's Hospital, and all patients gave written informed consent.

**Figure 1:**
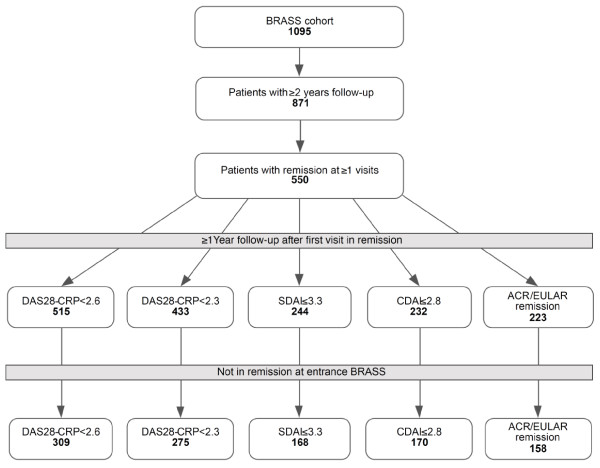
**Subject selection, illustrating the study cohort assembly**. For the Kaplan-Meier analyses, we include only subjects with at least 2 years of follow-up, at least one remission time-point with subsequently 12 months or more of follow-up, and who were not in remission at entrance into BRASS. BRASS, Brigham Rheumatoid Arthritis Sequential Study; DAS28-CRP, Disease Activity Score 28-C-reactive protein; ACR, American College of Rheumatology; SDAI, Simplified Disease Activity Index; CDAI, Clinical Disease Activity; EULAR, European League Against Rheumatism.

For the Kaplan-Meier analyses, we included only the 394 subjects with (a) at least 2 years follow-up, (b) at least one remission time-point with subsequently 12 months or more follow-up; and (c) were not in remission at entrance of BRASS (see Figure 1). The first remission time point for each patient was considered baseline (T = 0). Visits included ranged from March 2003 until June 2010.

### Remission criteria

The annually collected disease-activity variables were analyzed, and the proportion of patients in a state of remission was determined by the following criteria: DAS28-CRP4 < 2.6 and < 2.3, SDAI ≤3.3, CDAI ≤2.8, and the ACR/EULAR remission criteria. The remission criteria are described in detail in Table 1. The DAS28-CRP4 is based on a 28-joint tender and swollen count, patient's global assessment of disease activity, and the CRP [6]. Originally, remission threshold value for DAS28-CRP was set at 2.6, equal to the DAS28-ESR. However, because research has suggested that the cut-off values to classify remission are lower in DAS28-CRP compared with the DAS28-ESR, we also evaluated 2.3 as threshold value [15,16]. The SDAI and CDAI scores use the same variables as the DAS28-CRP (tender and swollen 28-joint count, patient's global assessment and CRP in the case of SDAI), but also include the physician's global assessment of disease activity [4,7]. The new ACR/EULAR remission criteria require that tender and swollen joint count, CRP (milligrams per liter), and patient's global assessment (on a 0 to 10 visual analogue scale) all individually to be ≤ 1 [8].

**Table 1:** Various definitions of remission in RA used in this study

Definition remission		Threshold of sum variables
DAS28-CRP4	0.56*√(TJC28) + 0.28*√(SJC28) + 0.36*ln(CRP+1) + 0.014*GH + 0.96	< 2.6
SDAI	TJC28 + SJC28 + CRP + PTglobal + MDglobal	≤3.3
CDAI	TJC28 + SJC28 + PTglobal + MDglobal	≤2.8
ACR/EULAR	TJC28* ≤1 & SJC28* ≤1 & CRP ≤1 & PTglobal ≤1	≤4

TJC28, 28 tender-joint count; SJC28, 28 swollen-joint count; CRP, C-reactive protein (mg/L DAS28-CRP4 and mg/dl other definitions); GH, General Health on a 0- to 100-mm Visual Analogue Scale (VAS); PTglobal, patient's global assessment on a 0- to 10-cm VAS;

MDglobal, physician's global assessment on a 0- to 10-cm VAS. *The ACR/EULAR definition prefers the inclusion of feet and ankles for the evaluation of remission, but use of the 28-joint count is allowed because the overall impact due to reduced joint count was found to be small.

### Statistical analyses

Descriptive statistics were reported as medians (interquartile range, IQR) for continuous variables and as frequencies (percentage) for categoric variables. The Mann-Whitney *U *test was used to compare continuous variables, and the Fisher Exact test for categoric variables. Patient and disease characteristics of the 224 patients with < 2 years of follow-up and not included in the study were compared with the 871 patients included in the study (≥2 years of follow-up).

The primary outcome was time in sustained remission according to the DAS28-CRP4, SDAI, CDAI, and ACR/EULAR criteria. The Kaplan-Meier curves and log-rank test were used to assess the difference of survival functions based on the five remission definitions. We separately calculated the number of subjects regaining remission for the DAS28-CRP < 2.6. Based on prior literature, we hypothesized that durability of remission may be different for subjects according to gender, serologic status (seropositive = rheumatoid factor (RF) or anti-cyclic citrullinated peptide (CCP) positive), and/or disease duration at start of remission [17-20]. Therefore, we evaluated subjects in sustained DAS28-CRP < 2.6 remission stratified according to these subgroups. We decided on a cut-off of 5 years for disease duration, because 0 to 5 years was the lowest quartile of disease duration of all patients at their first visit in remission (median, 11 years; IQR, 5 to 22).

For every RA patient in BRASS, the first time-point in remission according to one of the remission criteria was considered the baseline (T = 0) for that specific definition of remission. To calculate time in remission according to that definition, disease activity at consecutive annual time points was evaluated according to the remission criteria of that definition. After the subject no longer met the definition of a given remission criterion or was censored because of missing data, the data on the patient thereafter were ignored.

Apart from the survival analyses that censored patients who did not meeting the definition of remission, we also calculated the percentage of follow-up time spent in remission, allowing subjects to regain remission. For this secondary analysis, in contrary to the Kaplan-Meier analyses, we included all 871 subjects, regardless of whether they began BRASS follow-up in remission.

Nine percent of data on remission-criteria variables was missing. Multiple imputation by chained equations was performed to replace missing values in the variables requested for calculation of remission criteria [21]. Multiple imputation was applied only if a part of the remission variables were available; if all data were missing, the patient was considered lost to follow-up from that time on.

Outcomes were calculated by using statistical software SPSS 15.0.1. (SPSS Inc., Chicago, IL, USA) and SAS 9.2. (SAS Institute Inc., Cary, NC, USA).

## Results

### Patient and disease characteristics

Of the 1,095 RA patients in BRASS, 871 had at least 2 years of follow-up. Of the 871 subjects with at least 2 years of follow-up, 550 were in remission at one or more time-points. Of these subjects, 394 had 12 months of follow-up after their first remission time point and were not in remission at entrance into BRASS (see Figure 1). Median follow-up time for the cohort was 5.4 years (IQR, 4.5 to 6.5). Patient and disease characteristics at entrance into the BRASS cohort are given in Table 2. Patient and disease characteristics at entrance into BRASS of excluded subjects (< 2 years of follow-up) did not differ significantly from included subjects on median age, anti-CCP, and/or RF positivity, median CRP, morning stiffness, or use of medications. However, they did differ on gender (77% female; *P *= 0.049), smoking status (13% smokers; *P *= 0.029), median disease duration (7 years *P *= 0.024); use of MTX (35%; *P *= 0.001), and biologic DMARDs (28%; *P *= 0.006).

**Table 2:** Patient and disease characteristics at entrance into BRASS cohort for subjects with ≥2 years of follow-up, in remission at one or more visit to any remission definition, and not in remission at entrance

*N *= 394	*n *(%)/Median (IQR)
Female	320 (81)
Age (years)	56 (45-63)
Positive anti-CCP or RF status	282 (72)
Smoking	24 (6)
Disease duration (years)	8 (2-19)
Any stiffness	286 (73)
Stiffness duration (minutes)	30-60 (10-90)
CRP (mg/L)	2.7 (1.1-7.9)
NSAIDs	244 (62)
Corticosteroids	103 (26)
MTX	196 (50)
Nonbiologic DMARDs (not MTX)	137 (35)
Biologics	133 (34)

CCP, cyclic citrullinated peptide; CRP, C-reactive protein; DMARD, disease-modifying antirheumatic drug; MTX, methotrexate; RF, rheumatoid factor.

### Durability of sustained remission according to various remission definitions

**Figure 2:**
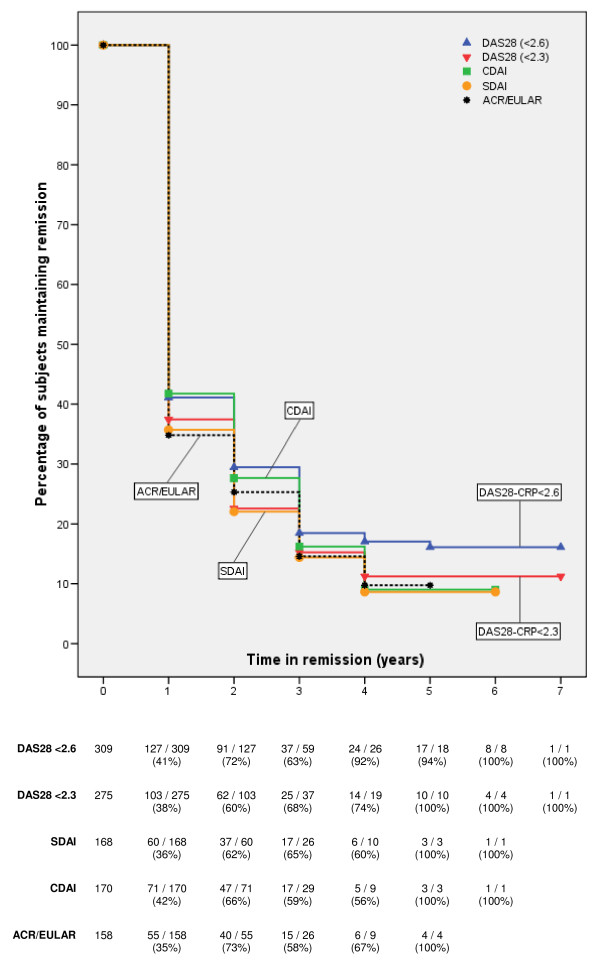
**Kaplan-Meier survival curves for subjects maintaining remission according to various remission definitions, demonstrating the Kaplan-Meier survival curves for the percentage of subjects maintaining remission over time**. Curves represent the following remission definitions: blue DAS28-CRP < 2.6 (blue), DAS28-CRP < 2.3 (red), CDAI (green), SDAI (yellow), and 2010 ACR/EULAR (black dotted). Beneath the figure, we include the number of subjects in remission and the percentage of subjects in remission compared with the total number of subjects included in the analysis at each time point. For instance, after 3 years, 37 of 59 patients (63%) were still in remission according to the DAS28-CRP < 2.6. Notice that not 91 but 59 subjects are evaluated at 3 years, because 32 subjects were censored, as not all patients have same duration of follow-up (open cohort).

The proportion of subjects remaining in remission according to the different remission criteria is shown in Figure 2. After 1 year, more than half of the RA patients previously in remission had fallen out of remission. Median remission survival, irrespective of the remission definition, was 1 year. The proportion of subjects considered to be in remission did not differ according to different remission criteria (log-rank test, *P *= 0.29). Remission using the DAS28-CRP at a 2.6 cut-off was met most frequently, and using SDAI and the new ACR/EULAR definition, least frequently.

One-hundred-eighty-two subjects had a median DAS28-CRP score of 3.8 (median tender-joint count of 6, swollen joint of 4, CRP of 6.6 mg/L, and general health on VAS of 25 mm) after they lost DAS28-CRP < 2.6 remission after one annual visit in remission. Of these 182 subjects who were in DAS28-CRP < 2.6 remission at one visit but not the subsequent annual visit, 41 (23%) regained remission at the next annual visit. Thirty-six subjects had a median DAS28-CRP score of 3.7 (median tender-joint count of 5, swollen joint of 4, CRP of 8.3 mg/L, and general health on VAS of 18 mm) after they lost DAS28-CRP < 2.6 remission after two subsequent annual visits in remission. Eleven (31%) of these 36 subjects who were in DAS28-CRP < 2.6 remission at two subsequent annual visits, but not the subsequent annual visit, regained remission at the next annual visit.

**Figure 3:**
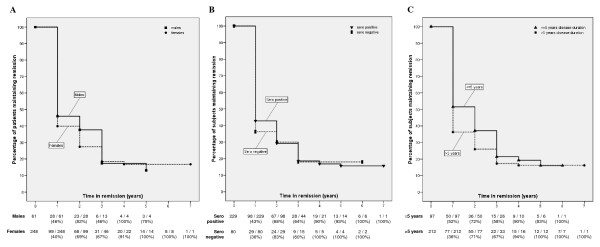
**Kaplan-Meier survival curves for subjects maintaining DAS28-CRP < 2.6 remission, stratified according to (a) gender, (b) serologic status, (c) disease duration**. We demonstrate the Kaplan-Meier survival curve for the percentage of patients maintaining DAS28-CRP < 2.6 remission over time. Patients are stratified according to different patient and disease characteristics. Beneath the figure, we include the number of subjects in remission and the percentage of subjects in remission compared with the total number of subjects included in the analysis at each time point.

In Figure 3, the Kaplan-Meier curves are shown for the 309 subjects in DAS28-CRP < 2.6 remission at ≥1 visit, stratified according to gender (Figure 3a), seropositivity (Figure 3b), and disease duration (Figure 3c). Although more males and subjects with ≤5 years of disease duration maintained remission in the first years of remission, the survival curves for these subgroups were not significantly different (log-rank test, *P *= 0.46 by gender, log-rank test, *P *= 0.12 by disease duration). Kaplan-Meier curves for seropositive and seronegative subjects were also similar (log-rank test, *P *= 0.92)

### Subjects according to percentage of visits in remission in BRASS

Considering all 871 subjects in BRASS with ≥2 years of follow-up, subjects on average spent 31% of their follow-up time in DAS28-CRP < 2.6 remission, 24% in DAS28-CRP < 2.3 remission, 12% in SDAI remission, 15% in CDAI remission, and 10% in ACR/EULAR remission. This calculation includes patients who were never in remission and subjects in remission at entrance into BRASS.

When we selected the 394 subjects who were in remission ≥1 visit and were not in remission at entrance into BRASS, the average time spent in remission was 48%, according to the DAS28-CRP < 2.6, 45% according to the DAS28-CRP < 2.3, 44% according to the SDAI, 44% according to the CDAI, and 42% according to the ACR/EULAR. The number of subjects according to mean time in remission and the various remission criteria are summarized in Table 3.

**Table 3:** Number of subjects categorized according to time spent in remission divided by subject-specific follow-up duration, for each of the remission criteria tested

All subjects in BRASS with ≥2 years of follow-up					
**Time in remission (%)**	**DAS28-CRP < 2.6** ***N *= 871^a^**	**DAS28-CRP < 2.3** ***N *= 871^a^**	**SDAI** ***N *= 871^a^**	**CDAI** ***N *= 871^a^**	**ACR/EULAR** ***N *= 871^a ^**

0	326 (37%)	401 (46%)	599 (69%)	610 (70%)	634 (73%)
1-25	130 (15%)	146 (17%)	94 (11%)	30 (3%)	87 (10%)
26-50	196 (23%)	163 (19%)	108 (12%)	127 (15%)	101 (12%)
51-75	121 (14%)	102 (12%)	52 (6%)	66 (8%)	39 (4%)
76-99	52 (6%)	30 (3%)	10 (1%)	17 (2%)	7 (1%)
100	46 (5%)	29 (3%)	8 (1%)	21 (2%)	3 (0.3%)

Overall mean time in remission	31%	24%	12%	15%	10%

**Selected population of subjects in BRASS**					

**Time in remission (%)**	**DAS28-CRP < 2.6** ***N *= 309**^b^	**DAS28-CRP < 2.3** ***N *= 275**^b^	**SDAI** ***N *= 168**^b^	**CDAI** ***N *= 170**^b^	**ACR/EULAR** ***N *= 158**^b^

1-25	77 (25%)	89 (32%)	58 (35%)	54 (32%)	60 (38%)
26-50	133 (43%)	111 (40%)	62 (37%)	72 (42%)	64 (41%)
51-75	49 (16%)	37 (13%)	30 (18%)	24 (14%)	18 (11%)
76-99	28 (9%)	21 (8%)	6 (4%)	7 (4%)	8 (5%)
100	22 (7%)	17 (6%)	12 (7%)	13 (8%)	8 (5%)

Overall mean time in remission	48%	45%	44%	44%	42%

**^a^**Subjects in BRASS with ≥2 years of follow-up. Maximum number of visits is eight. ^b^Subjects in BRASS with ≥2 years of follow-up, one or more visits in remission, according to the various

definitions; ≥1 year follow-up after first visit in remission and not in remission at entrance into

BRASS. Maximum number of visits is seven (because visit at entrance into BRASS is excluded).

## Discussion

Because of advances made in therapy and treatment strategies, remission in RA has become the treatment goal in clinical trials and in clinical practice. However, previous studies have already suggested that remission in clinical practice might not be as common as in clinical trials [22,23]. The aim of our study was to evaluate whether subjects in remission maintain this state over a long period. Our analyses show that not even half of the RA patients maintained remission beyond 1 year, regardless of the remission definition (see Figure 1). Even after multiple years in remission, a patient's disease can become more active. These findings have clinical implications, in that physicians need to continue to monitor RA patients in remission closely and may need to consider treatment changes in these patients if they flare or present with sustained disease activity. According to the Kaplan-Meier survival-curve analyses, the likelihood of patients experiencing active disease after remission decreases as the years in remission increase. Whether patients were considered to be in remission depended on the criteria used, although differences in remission duration were not statistically significant. We found the SDAI and the new ACR/EULAR criteria to be the most stringent. This is in accordance with the aims of the ACR, EULAR, and OMERACT, who requested a stringent remission definition and proposed the SDAI and newly developed ACR/EULAR remission [9]. Although the ACR/EULAR remission definition was designed and validated for use in clinical trials and not for observational studies evaluating remission in real-life clinical practice, we thought it would be interesting to evaluate the definition in the current study [8-11].

It should be noted that aging of patients could have played a role in not fulfilling remission criteria, because subjects had a median age of 56 years at entrance to BRASS. A study from Finland evaluated individuals from the general population older than 50 years by the ACR remission criteria and concluded that the majority did not meet the criteria [24]. However, the aim of the current study was to evaluate durability of remission, not the incidence of remission. It is not likely that age could have played a major role in the fact that more than half of subjects did not meet remission criteria any more after only 1 year.

Other studies have described sustained remission in daily practice as uncommon, being reached by only 17% to 36% of RA patients for up to 6 months [19,22,25,26]. These studies did not evaluate time in remission beyond 6 months. A recent study investigated the probability of remaining in remission up to 24 months, according to the ACR/EULAR, SDAI, and CDAI remission criteria in two different cohorts [27]. They also concluded that long-term remission is rare, considering that the probability of a remission lasting 2 years was 6% to 14%.

We evaluated differences in duration of sustained remission according to patient and disease characteristics. We did not find statistically significant differences based on gender, seropositivity, or disease duration, but these stratified analyses were underpowered and should be considered only exploratory. A trend seemed to exist toward longer remission duration in men and subjects with 5 years or less disease duration at start of remission. Schipper *et al. *[19] recently showed that time to achieve remission correlates with remission duration [19]. Although our data suggest the same, we cannot be sure because most BRASS patients enter the study after the initiation of disease; thus subjects could have been in remission before entrance into BRASS.

In addition to data on remission duration, we investigated patterns of regaining remission for DAS-CRP < 2.6. Fewer than a quarter of subjects who were previously in remission at one visit, and then dropped out of remission, regained remission at the next annual visit. Slightly more subjects regained remission if demonstrating remission at two consecutive visits, but the percentage was still low (31%). Subsequently, we evaluated time spent in remission during the specific follow-up period for each subject in BRASS with at least 2 years of follow-up. These data not only show that sustained remission is uncommon in clinical practice, but also that most subjects spend little follow-up time in remission. In the BRASS cohort, most subjects were in remission during less than half of their follow-up visits.

The ultimate goal of treating RA is to achieve remission and halt the progression of joint damage. Several studies show that one time point of meeting remission criteria does not always mean that the disease does not progress [28-30]. A longer period of meeting the remission criteria might be an indication of cessation of disease activity, rather than drug-induced disease suppression [31]. In juvenile idiopathic arthritis, patients with inactive disease at two or more yearly visits developed less cumulative joint damage than did those in such a state at only one visit [32]. These data suggest that it is important not only to reach remission, but also to maintain this state. It will be important to determine whether RA patients in sustained remission have less disease destruction compared with those not in sustained remission.

In a recently published study, we showed that an increased number of visits in remission was associated with reduced radiographic damage [30]. The goal of the current study was to describe the duration of remission with different criteria; we did not examine how patients achieved remission in this study. This will be the focus of future work.

The BRASS cohort represents patients from one academic practice setting, and data are gathered over a long period. In this single-center cohort, most RA patients entered the cohort with longstanding disease. Because disease activity was measured yearly in BRASS, it is possible that a patient's disease flared between visits. Thus, this limitation of our data would strengthen the conclusion that one time point of remission in no guarantee for sustained remission. Also this will not affect the comparisons of different remission criteria in the survival analysis, because all remission criteria were measured at similar time points.

## Conclusion

Recently published recommendations for the management of RA by EULAR suggest that as long as a target of remission or low disease activity has not been reached, treatment should be adjusted by frequent and strict monitoring [2]. Although remission without respect to duration is a good goal, it is not the most important target for therapy. Data from our study confirm that even after reaching remission, strict monitoring is required, as fewer than half of the patients in clinical practice will not sustain remission for more than 1 year.

## Abbreviations

ACR: American College of Rheumatology; BRASS: Brigham Rheumatoid Arthritis Sequential Study; CDAI: Clinical Disease Activity Index; CRP: C-reactive protein; DAS: Disease Activity Score; ESR: erythrocyte sedimentation rate; EULAR: European League Against Rheumatism; IQR: interquartile range; OMERACT: Outcome Measures in Rheumatology Initiative; RA: rheumatoid arthritis; SDAI: Simplified Disease Activity Index.

## Competing interests

Dr Femke Prince has received financial support for educational purposes from Abbott, Bristol-Myers Squib, Novartis Pharma, Teva Pharma, and Wyeth Pharmaceuticals, and a consulting fee from Bristol-Myers Squib. Dr Nancy Shadick has received research grant support from Amgen, Crescendo Bioscience, Biogen Idec, Genentech, Abbott, and Medimmune. Prof. Michael Weinblatt has received grant support from Biogen Idec, Crescendo Bioscience, and consultant fees from Biogen Idec and Crescendo Bioscience. Dr Daniel Solomon has received financial support for educational purposes from Bristol-Myers Squibb and research grants from Abbott Immunology and Amgen.

## Authors' contributions

FP contributed to the design of the study, conducted the initial statistical analyses, contributed to the interpretation of data, and wrote the first draft of the manuscript. VP, NS, CI, and MW contributed to the design of the study and interpretation of data. BL, JC, and MF contributed to the management and analysis of data. DS contributed to the design of the study, analysis and interpretation of data, and helped to write the first draft of the manuscript. All authors critically reviewed, contributed to, and approved the final manuscript.
